# Metabolic Alterations in Women with Lipedema Compared to Women with Lifestyle-Induced Overweight/Obesity

**DOI:** 10.3390/biomedicines13040867

**Published:** 2025-04-03

**Authors:** Małgorzata Jeziorek, Maciej Wuczyński, Monika Sowicz, Agnieszka Adaszyńska, Andrzej Szuba, Angelika Chachaj

**Affiliations:** 1 Department of Dietetics and Bromatology, Faculty of Pharmacy, Wroclaw Medical University, 50-367 Wroclaw, Poland; 2 Statistical Analysis Centre, Wroclaw Medical University, 50-372 Wroclaw, Poland; maciej.wuczynski@umw.edu.pl; 3 Department of Angiology and Internal Medicine, Wroclaw Medical University, 50-367 Wroclaw, Poland; monika.sowicz@wp.pl (M.S.); agnieszka.adaszynska@usk.wroc.pl (A.A.); andrzej.szuba@umw.edu.pl (A.S.); angelika.chachaj@umw.edu.pl (A.C.)

**Keywords:** lipedema, overweight/obesity, metabolic alterations, fat distribution

## Abstract

Background: Lipedema is a chronic disorder characterized by disproportionate fat accumulation in the extremities, predominantly affecting women. Unlike obesity, lipedema might be associated with a lower prevalence of metabolic alterations despite often coexisting with overweight or obesity. Fat distribution plays a crucial role in metabolic differences, with gynoid fat in lipedema being associated with a lower risk of insulin resistance and cardiovascular disease. The present study aims to compare biochemical parameters between women with lipedema and those with lifestyle-induced overweight/obesity. **Methods**: This study involved 108 women, including 53 with lipedema and 55 with lifestyle-induced overweight/obesity. Anthropometric measurements and body composition analyses were conducted, and biochemical parameters related to liver function, lipid profile, kidney and thyroid function, uric acid levels, and carbohydrate metabolism were assessed. Statistical comparisons were made between groups, and multivariate models were used to explore the relationships between disease type, metabolic parameters, and body composition. **Results**: Women with lipedema exhibited a more favorable metabolic profile than those with overweight/obesity. Dyslipidemia was observed in ~50% of the lipedema group, compared to nearly 70% in the overweight/obesity group. Impaired glucose metabolism and insulin resistance were significantly less prevalent in the lipedema group (18.9% vs. 43.6%, *p* < 0.05). **Conclusions**: Despite having a high BMI, women with lipedema demonstrate fewer metabolic alterations than those with overweight/obesity. Fat distribution, rather than overall adiposity, appears to influence metabolic risk. These findings highlight the need for targeted therapeutic approaches to lipedema, distinct from conventional obesity management strategies.

## 1. Introduction

Lipedema is a chronic disease characterized by excessive fat accumulation in the legs, and, sometimes, in the arms. The typical clinical symptoms of lipedema an imbalance between the minor upper and more extended lower body, pressure-induced pain in the legs, and easy bruising [1]. Overweight or obesity co-occurs in approximately 80% of patients with lipedema [2,3]. Lipedema is a relatively common disease that occurs almost exclusively in women and may affect nearly 11% of the global population [4,5]. The disease, especially in advanced stages, can lead to psychosocial consequences, including social isolation or depression [3]. The etiology of lipedema is unknown, but genetic, hormonal, vascular, and inflammatory factors are hypothesized to be involved in the pathogenesis of the disease [6]. Typical calorie-restricted diets with physical activity or bariatric surgery are not effective treatments for lipedema [6,7]. Because lipedema is characterized by low-grade inflammation in fatty tissue, researchers indicate that a low-calorie diet rich in anti-inflammatory products (such as the Mediterranean diet) might be beneficial in treating the disease [8,9]. Some studies have observed reduced subcutaneous fatty tissue in the legs due to a ketogenic diet [10,11,12,13]. Our previous studies demonstrated a significant decrease in body mass, a reduction in fatty tissue in the legs, and a reduction in pain after 7 months of an anti-inflammatory modified Mediterranean ketogenic diet in lipedema patients [14].

Despite lipedema being a chronic condition characterized by excessive fat accumulation, research indicates that metabolic alterations occur less frequently in patients with lipedema than in those with obesity [15]. Women with lipedema may have a better lipid and glucose profile, less common insulin resistance (IR), and a lower risk of cardiovascular disease (CVD) compared to women with overweight or obesity. These differences are likely linked to the location of fat accumulation. In obesity, fat is often concentrated in the abdomen, leading to an increase in proinflammatory cytokines and a higher risk of metabolic diseases. In contrast, lipedema primarily involves fat accumulation in the extremities (gynoid fat) [4,16]. Research is required to explore the impact of fat distribution on metabolic disorders in patients with lipedema. To the best of our knowledge, this will be the first study examining this issue. The results of this study will be significant for acquiring knowledge about the general health status of patients with lipedema and will be helpful in directing further scientific efforts towards understanding the nature of the disease. They may also serve as a basis for developing more effective therapeutic strategies for individuals suffering from this condition.

The aim of this cross-sectional study was the comparative analysis of various biochemical parameters, namely liver function, lipid profile, kidney and thyroid function, uric acid level and carbohydrate metabolism, in female patients with lipedema and women with lifestyle-induced overweight/obesity without lipedema.

## 2. Materials and Methods

### 2.1. Study Population

The study involved 108 women, including 53 diagnosed with lipedema by an angiologist based on typical symptoms [17] and 55 who were overweight or obese with a BMI greater than 25 kg/m^2^ but who did not have lipedema. All participants were recruited from the Angiology Outpatient Clinic at Wroclaw Medical University in Poland between January 2021 and May 2022. The exclusion criteria for all study subjects included being pregnant, breastfeeding, being six months post-pregnancy, having a diagnosis of lymphedema, exhibiting edema due to chronic venous insufficiency or heart failure, and having diabetes mellitus, kidney or liver failure, hormonally unbalanced thyroid disease, cancer, the presence of implanted cardiac devices (such as a pacemaker, implantable cardioverter-defibrillator, or resynchronization therapy) or metal implants. Patients in the lipedema group were categorized into 3 stages. Stage 1 featured smooth skin over small nodules in a thickened fat layer, stage 2 featured skin indentation over larger fat masses, and stage 3 involved both small nodules and larger fat masses, creating skin and fat lobules, primarily on the hips, thighs, and knees [17]. The study was conducted in compliance with the Declaration of Helsinki, and all procedures involving human subjects were approved by the Bioethics Committee guidelines at Wroclaw Medical University, Poland (KB—690/2017) on 23 November 2017. Written informed consent was obtained from all participants.

### 2.2. Body Composition and Anthropometric Parameters

A TANITA HR-001 growth meter (Tanita, Tokio, Japan) was used to measure height, while a TANITA MC-780MA (Tanita, Japan) was utilized to determine weight and body composition parameters, including PBF (body fat percentage, %), MBF (body fat, kg), LBM (lean body mass, kg), TBW (total body water, kg) and VFL (visceral fat level), using bioimpedance analysis. The Body Mass Index (BMI, kg/m^2^) was calculated as the ratio of body weight in kilograms to the square of height in meters. Waist, hip, thigh, calf, and ankle circumferences were measured using a standard tape measure with a precision of up to 1 cm. The waist/hip ratio (WHR) was determined by dividing the waist by the hip circumference. Leg circumferences were measured with a standard tape measure to the nearest 0.5 cm. The measurements were taken at 4 cm intervals from the ankle to the groin on the side of the leg. Based on these measurements, we calculated the volume of the leg (in milliliters) using the equation for a truncated cone [18].

### 2.3. Blood Samples

Blood samples were collected after 12 h of fasting in both groups. The parameters analyzed included bilirubin, aspartate aminotransferase (AST), alanine transaminase (ALT), gamma-glutamyl transpeptidase (GGTP), alkaline phosphatase (ALP), total cholesterol (TC), low-density lipoprotein cholesterol (LDL-C), high-density lipoprotein cholesterol (HDL-C), triglycerides (TG), creatinine, the glomerular filtration rate (GFR), uric acid, thyroid-stimulating hormone (TSH), glycated hemoglobin (HbA1c), glucose, and insulin levels. A three-point oral glucose tolerance test (OGTT) using a 75 g glucose solution was also performed. The Homeostatic Model Assessment for Insulin Resistance (HOMA-IR) was calculated as the ratio of fasting glucose [mg/dL] and insulin [μU/mL] to a constant value of 405 [19].

### 2.4. Statistical Analysis

Means and standard deviations were calculated for each parameter characterizing the study group. Comparisons were made to check for differences between lipedema and overweight/obese patients. In order to test for the significance of differences between groups, the following procedure was carried out. The Kolmogorov–Smirnov test was used to check if the data in both groups were normally distributed. The Levene test was used to check for the homogeneity of variances. If the data in groups were normally distributed and with similar variances, Student’s *t*-test was performed to compare the groups. If the data were normally distributed, but two distributions had unequal variances, Welch’s *t*-test was performed. If the data in at least one group were non-normally distributed, the Mann–Whitney U-test was used to check differences between groups. Next, the prevalence of metabolic alterations was compared between the two groups using the Pearson’s Chi-Square Independence Test with Yates’s correction. In all tests, the significance level equaled 0.05.

Given the large number of biochemical and anthropometric predictors, we hypothesized that there might be some correlations between the biochemical parameters and specific study groups, as well as their anthropometric and body composition parameters. In order to test for the relation between disease type and metabolic alterations, a series of Generalized Linear Models was fitted to each target variable. Due to the binary nature of targets, a logit model was chosen. The high number of numerical predictors (13) was reduced using Principal Component Analysis (PCA) with orthogonal rotation, which retained 87% of data variability in 3 components, called PC1–PC3. The number of components was chosen visually using a scree plot (Supplementary Material, Figure S1) and numerically using Kaiser rule, i.e., dropping components with less variability than a single variable in the original dataset (Supplementary Material, Table S1). The coordinates of selected components were not transformed, as popular transformations (varimax and quartimax) did not improve the interpretability or significance of the results. Additionally, the disease type and its interactions with PCs were used as predictors. A complete set of possible predictor combinations was calculated and ranked for each target variable using the Akkaike Criterion (AICc). A subset of the best models was then selected by selecting models with AICc delta < 2 (Supplementary Material, Table S2). This subset was averaged, resulting in a weighted estimate that is presented in Table S3 (Supplementary Material). In the same set of tables, a relative variable importance (RVI) is presented as the number of presences in the subset and as a sum of weights [20]. A summary of the modelling results is presented in the results section. Original predictors include disease type (lipedema and overweight/obesity), age, height, body mass, BMI, LBM, PBF, MBF, TBW, VFL, waist, hip, WHR, and average leg volume. The final predictors include disease type (reference: lipedema), PC1, PC2, PC3, PC1 × Disease type, PC2 × Disease type and PC3 × Disease type.

All calculations were performed in R 4.4.1 programming language. Group comparisons were made using t.test and wilcox.test functions. Models were calculated using the glm function from the lme4 1.1.35.5 library; then, they were selected and averaged using dredge and model.avg from MuMIn 1.48.4. Principal Component Analysis was performed using the principal function from the psych 2.4.6.26 package.

## 3. Results

The majority of patients with lipedema were in stage 2 (56.6%), followed by stage 1 (37.7%) and stage 3 (5.7%). The overweight/obesity group consisted of 25.5% women with overweight (BMI 25–29.9 kg/m^2^), 27.3% with class 1 obesity (BMI 30–34.9 kg/m^2^), 25.4% with class 2 obesity (BMI 35–39.9 kg/m^2^), and 21.8% with morbid obesity (BMI ≥ 40 kg/m^2^). Table 1 presents the general characteristics of the anthropometric and body composition parameters, as well as the leg circumferences. Patients differed significantly in weight, BMI, LBM, PBF, MBF, TBW, and WHR. The visceral fat levels measured by BIA did not differ significantly between the study groups and indicated slightly worse results in the lipedema group. However, overweight/obese patients had significantly higher waist measurements and WHR, indicating more visceral fat (central obesity) in this group.

Liver parameters (ALT and GGTP) and kidney parameters (creatinine and GFR), as well as uric acid levels, were significantly better in the lipedema group compared to the overweight/obesity group (Table 2). Alterations in liver enzymes (ALT, AST), HDL-C levels, uric acid, and glucose levels were similarly prevalent in both groups; however, these changes were less pronounced in the lipedema group (Table 3). In this study, the mean values of the lipid parameters, including LDL-C, HDL-C, and TG, were significantly lower in the lipedema group compared to the overweight/obesity group (Table 2). Hypertriglyceridemia was more common in the overweight/obesity group than in the lipedema group (25.5% vs. 5.7%; *p* = 0.012). While hypercholesterolemia was more frequent in the overweight/obesity group than in the lipedema group (67.3% vs. 49.1%), this difference was not statistically significant (*p* = 0.106) (Table 3). When analyzing carbohydrate metabolism disorders, HbA1c [%] was slightly lower in the lipedema group compared to the overweight/obesity group; however, it remained within the normal range in both groups. Impaired fasting glucose and glucose tolerance were more pronounced in the overweight/obesity group. In this study, insulin resistance was observed in less than 12% of women with lipedema, compared to nearly 35% of women with overweight/obesity (*p* = 0.01) (Table 3).

The higher occurrence of all these metabolic alterations in the overweight/obesity group seems evident due to the significantly higher weight and BMI in this group. However, the impact of fat distribution on metabolic alterations in patients with lipedema remains unexplored.

Table 4 shows the relationships between three principal components (PC1, PC2, PC3) and various body composition parameters. PC1 was strongly associated with weight (0.35), BMI (0.34), MBF (0.35), and waist/hip measurements (~0.33), representing the overall body size, including both fat and lean mass. PC2 was strongly negatively related to height (−0.54) and positively related to age (0.50). This likely captures age and height differences, distinguishing taller/younger individuals from shorter/older ones. PC3 was strongly positively related to VFL (0.41) and the average leg volume (0.40), and strongly negatively related to WHR (−0.62). This likely represents the patterns of fat distribution, differentiating between visceral fat and extremity fat.

A greater body size (PC1) generally had a negative impact on overall health. Patients with overweight/obesity were at a higher risk of out-of-range results, and the combined effects of these factors led to even worse outcomes. Body fat distribution (PC3) was a significant predictor with mixed effects in the models, sometimes associated with better results and at other times with worse outcomes. PC2 (higher age and lower height) consistently had a negative impact; however, PC2 appeared less frequently in the models, which resulted in its weaker overall influence (Table S3 in Supplementary Materials). Overweight/obese patients were more likely to have abnormal ALT levels. A higher PC3, indicating more fat in the visceral area, was also linked to worse ALT levels. However, this effect was less pronounced in overweight/obese patients, likely because obesity already strongly affects liver health. The AST models were ineffective, likely due to highly imbalanced data, making it hard to identify meaningful patterns. Older patients had a higher chance of LDL-C level alterations. At the same time, those with a higher PC3 had slightly better LDL-C. The effect of PC3 on LDL-C was more pronounced in overweight/obese individuals, suggesting that it is linked to visceral obesity and the protective role of fat in the extremities. The interaction between PC3 and the metabolic alteration type influenced some models, indicating that the relationship between PC3 and LDL-C depends on the disease type (lipedema vs. overweight/obesity). Connections between the disease type and HDL-C levels were observed in only one model, with a modest effect size. Worse triglyceride results were clearly associated with a higher general body size (PC1) and lower PC3, which indicates that individuals with less fat in the extremities (but more visceral fat) had worse triglyceride levels. The same two predictors had a pronounced impact on uric acid levels. Patients with a larger body size (PC1) had worse fasting glucose levels, and overweight/obese patients generally had slightly worse results compared to others. More fat in the extremities (vs. abdomen) was associated with better glucose levels, especially in non-obese patients. Older patients generally more frequently had impaired glucose tolerance on average. Overweight/obese patients generally showed worse outcomes regarding insulin resistance (HOMA-IR), especially as their body size (PC1) increased (Table S3 in Supplementary Materials).

Table 5 shows the averaged RVI (Relative Variable Importance), which represents the importance of various predictors in explaining the target variables. We observed that PC3 is the most significant predictor of metabolic alterations, even stronger than body size itself (PC1). Table 6 summarizes the direction of estimated effects for averaged models across various variables (e.g., ALT, LDL-C, etc.) in relation to disease (overweight/obesity), principal components (PC1–PC3), and their interactions with disease.

## 4. Discussion

Lipedema, a chronic condition characterized by excessive fat accumulation in the extremities, differs metabolically from obesity. Research suggests that metabolic complications are less common in individuals with lipedema than in those with obesity. Women with lipedema tend to exhibit a better lipid and glucose profile, a lower prevalence of insulin resistance, and a reduced risk of cardiovascular disease compared to women who have lifestyle-induced overweight/obesity (but do not have lipedema), despite most of them also being characterized by overweight or obesity. This might be explained by the fact that in lipedema, fat predominantly accumulates in the extremities (gynoid fat). In contrast, obesity is often associated with visceral fat concentrated in the abdominal region [15]. An increased waist circumference, indicative of abdominal obesity, is strongly linked to a higher risk of metabolic dysfunctions such as diabetes, cardiovascular disease, and hypertension. Furthermore, visceral fat not only acts as a marker for these risks but also plays a direct role in their development, making waist circumference an essential clinical measure for identifying individuals at risk [21,22].

We found that fat distribution plays a crucial role in the development of metabolic diseases when comparing the lipedema group with individuals who are overweight/obese. This effect appears to be even stronger than body size itself. Our findings suggest that a greater proportion of fat in the extremities (as opposed to visceral fat) may have a protective impact on overall metabolic health, particularly in terms of LDL-C, TG, glucose, and insulin resistance, regardless of the patient’s weight.

In this study, dyslipidemia was observed in approximately 50% of women with lipedema, compared to nearly 70% of patients with overweight or obesity. Notably, hypertriglyceridemia was more prevalent in the overweight/obesity group. These findings partially align with those reported by other authors, particularly the conclusion that lipid alterations are more frequent in individuals with obesity than in those with lipedema. However, we also observed a notable prevalence of lipid alterations in patients with lipedema. In a study conducted by Torre et al. [15], the majority of women with lipedema had a normal lipid profile, with only 11.7% showing dyslipidemia, compared to 33.5% of females in the general population and even higher percentages among obese women. Similarly, Herbst et al. [23] reported that dyslipidemia occurred in 38% of female lipedema patients in their study (*n* = 50). The authors hypothesized that, in its earlier stages, lipedema fat is distributed in a predominantly gynoid pattern (hips, buttocks, and thighs), which is associated with a lower cardiovascular risk. Additionally, a study assessing comorbidities in patients with lipedema found that 27% of the patients (*n* = 15) were characterized by dyslipidemia [24]. Supporting this, Mekki et al. [25] found that women with a gynoid fat distribution had lower triglyceride levels compared to those with an android fat distribution. This suggests that gynoid fat may protect against abnormal blood lipid levels, which are typically associated with increased cardiovascular risk. Gynoid fat is believed to be associated with a lower risk of cardiovascular disease (CVD) compared to abdominal fat, which is more common in lifestyle-induced obesity. Incorporating body shape alongside weight has been shown to provide a more accurate estimate of morbidity risk than weight alone. Consequently, women with lipedema may have a reduced CVD risk due to the predominance of gynoid fat [15].

Patients with lipedema are reported to have a lower risk of insulin resistance and diabetes compared to individuals with obesity [15]. In this study, the prevalence of carbohydrate metabolism alterations, including impaired glucose fasting, impaired glucose tolerance and insulin resistance, was observed in 18.9% of patients in the lipedema group, compared to 43.6% in the overweight/obesity group. Insulin resistance was three times less frequent in the lipedema group than in the overweight/obesity group (11.3% vs. 34.5%; *p* = 0.01). Faerber [22] noted that obese patients with lipedema exhibited lower levels of insulin resistance compared to a control group of obese individuals without lipedema, despite having a higher average weight. However, this difference diminished as abdominal obesity increased. In a study by Wanshu et al. [24], diabetes or glucose intolerance was observed in 13% of patients with lipedema (*n* = 15). Similarly, Pinnick et al. [26] demonstrated that gynoid fat negatively correlates with insulin resistance after adjusting for total fat, whereas abdominal fat shows the opposite trend. This suggests that lipedema fat may provide a protective effect against diabetes. A study by Rasmussen et al. [27] found that, while M1 macrophages are typically prevalent in the adipose tissue of individuals with obesity, lipedema fat is characterized by a higher proportion of anti-inflammatory, M2-polarized macrophages. This difference may partially explain the absence of insulin resistance and the relatively low risk of diabetes in individuals with lipedema. Herbst et al. [23] reported a very low incidence of type 2 diabetes (2%, *n* = 50), suggesting that lipedema fat may not be insulin-resistant, or that patients with lipedema are protected from diabetes through other mechanisms. In a separate study, the same author concluded that, despite an elevated BMI, the incidence of diabetes among women with lipedema remains relatively low [28] Data presented by Torre et al. [15] indicate that the risk of diabetes in patients with lipedema is low (~2%), for an average BMI of 35.3 ± 1.7 kg/m^2^. Nonetheless, the risk of pre-diabetes appears to increase in Stages 2 and 3 of lipedema, suggesting that the likelihood of developing diabetes may rise as the condition progresses [15]. However, further research is needed to investigate this phenomenon in larger cohorts of lipedema patients and to clarify the underlying mechanisms.

The main strength of the study is the well-matched pairing of women with lipedema and those with obesity with regard to their age, allowing for a direct comparison of their metabolic profiles. This approach provides valuable and novel insights into identifying the risk factors associated with the development of cardiovascular diseases and preventive strategies. Another strength is the comprehensive biochemical assessment, as well as the novelty and clinical relevance of the findings, highlighting the need for distinct therapeutic strategies for lipedema, separate from conventional obesity management. The limitations of this study include the fact that the cross-sectional nature of the study captures only a single time point. Additionally, the relatively small sample size and monocentric design may limit the generalizability of the findings to broader populations. Furthermore, there is a lack of ethnic diversity, as the study focuses solely on white women.

## 5. Conclusions

This study highlights that fat distribution plays a crucial role in metabolic health, with women with lipedema exhibiting a more favorable metabolic profile than those with lifestyle-induced overweight or obesity. The predominance of gynoid fat in lipedema, as opposed to visceral fat in obesity, appears to have a protective effect against metabolic disorders, including cardiovascular disease and diabetes.

## Figures and Tables

**Table 1:** Characteristics of the anthropometric and body composition parameters, and leg circumferences.

Parameter	Lipedema Mean ± SD	Overweight/ Obesity Mean ± SD	*p*-Value
Age [years]	42.04 ± 14.22	44.80 ± 12.95	0.163 *
Height [cm]	165.80 ± 7.46	165.82 ± 6.24	0.990 ^
Weight [kg]	86.35 ± 20.63	98.13 ± 20.69	0.004 ^
BMI [kg/m^2^]	31.53 ± 7.53	35.59 ± 6.85	0.004 ^
LBM [kg]	51.80 ± 8.51	55.20 ± 8.63	0.042 ^
PBF [%]	37.77 ± 7.31	40.45 ± 4.95	0.029 ’
MBF [kg]	33.75 ± 13.21	40.45 ± 12.87	0.009 ^
TBW [kg]	38.65 ± 6.48	41.34 ± 6.44	0.033 ^
VFL	11.81 ± 5.24	11.07 ± 4.28	0.423 ^
Waist [cm]	97.19 ± 15.40	111.12 ± 15.30	<0.001 ^
Hip [cm]	114.50 ± 13.71	118.21 ± 13.58	0.161 ^
WHR	0.85 ± 0.09	0.94 ± 0.07	<0.001 ’
Left thigh [cm]	65.60 ± 8.03	66.73 ± 6.94	0.438 ^
Right thigh [cm]	65.57 ± 8.08	66.79 ± 6.99	0.401 ^
Left calf [cm]	44.74 ± 5.83	42.87 ± 4.66	0.069 ^
Right calf [cm]	44.49 ± 5.72	42.87 ± 4.75	0.112 ^
Left ankle [cm]	24.96 ± 2.74	23.97 ± 2.59	0.056 ^
Right ankle [cm]	24.61 ± 2.62	24.07 ± 2.80	0.095 *
Volume right leg [mL]	13,408.98 ± 5726.75	11,577.05 ± 2674.02	0.038 ^
Volume left leg [mL]	12,951.33 ± 3172.84	11,912.23 ± 2802.43	0.081 ^

BMI—body mass index; LBM—lean body mass; PBF—percentage body fat; MBF—mass of body fat; TBW—total body water; VFL—visceral fat level; WHR—waist/hip ratio; * Mann–-Whitney test; ^ Student’s *t*-test; ’ Welch test.

**Table 2:** Characteristics of biochemical parameters in study groups.

Parameter	Lipedema Mean ± SD	Overweight/Obesity Mean ± SD	*p*-Value
Bilirubin [mg/dL]	0.65 ± 0.24	2.16 ± 11.76	0.096 *
AST [U/L]	23.38 ± 6.20	25.22 ± 7.09	0.158 ^
ALT [U/L]	20.54 ± 10.34	26.89 ± 13.50	0.001 *
GGTP [U/L]	19.84 ± 12.00	25.17 ± 15.19	0.008 *
ALP [U/L]	64.10 ± 19.90	68.23 ± 21.18	0.314 ^
TC [mg/dL]	205.48 ± 39.10	221.40 ± 46.68	0.059 ^
LDL-C [mg/dL]	119.04 ± 30.05	138.47 ± 45.94	0.011 ’
HDL-C [mg/dL]	67.00 ± 16.98	58.95 ± 12.50	0.006 ^
TG [mg/dL]	89.88 ± 45.54	121.98 ± 66.08	0.004 ’
Creatine [mg/dL]	0.81 ± 0.13	0.84 ± 0.10	0.024 *
GFR [mL/min/1.73 m^2^]	86.98 ± 14.14	80.26 ± 13.47	0.014 ^
Uric acid [mg/dL]	4.75 ± 1.40	5.43 ± 1.19	0.009 ^
TSH [µIU/mL]	1.98 ± 1.40	2.05 ± 2.05	0.633 *
HbA1c [%]	5.29 ± 0.36	5.59 ± 0.74	0.001 *
Glucose 0 min. [mg/dL]	85.47 ± 16.95	90.95 ± 23.35	0.054 *
Glucose 60 min. [mg/dL]	120.10 ± 41.85	150.80 ± 57.29	0.005 ^
Glucose 120 min. [mg/dL]	100.33 ± 35.06	114.17 ± 41.61	0.094 ^
Insulin 0 min. [µU/mL]	8.74 ± 11.30	10.09 ± 5.35	0.002 *
Insulin 60 min. [µU/mL]	50.02 ± 39.56	74.53 ± 46.71	0.009 ^
Insulin 120 min. [µU/mL]	31.55 ± 28.36	50.71 ± 43.15	0.015 *
HOMA-IR	1.90 ± 2.55	2.36 ± 1.64	0.002 *

AST—aspartate aminotransferase; ALT—alanine transaminase; GGTP—gamma-glutamyl transpeptidase; ALP—alkaline phosphatase; TC—total-cholesterol, LDL-C—low-density lipoprotein cholesterol; HDL—high-density lipoprotein cholesterol; TG—triglycerides; GFR—glomerular filtration rate; TSH—thyroid stimulating hormone; HbA1c—glycated hemoglobin; HOMA-IR—Homeostatic Model Assessment for Insulin Resistance; * Mann–Whitney test; ^ Student’s *t*-test; ’ Welch test.

**Table 3:** Prevalence of metabolic alterations in lipedema (*n* = 53) and overweight/obesity (*n* = 55) group.

Parameter	Lipedema *n* (%)	Overweight/Obesity *n* (%)	*p*-Value
ALT (>35 U/L)	4 (7.5%)	9 (16.4%)	0.282
AST (>31 U/L)	7 (13.2%)	8 (14.5%)	1.000
Hypercholesterolaemia (LDL-C ≥ 115 mg/dL)	26 (49.1%)	37 (67.3%)	0.106
HDL < 45 mg/dL	2 (3.8%)	6 (10.9%)	0.307
Hypertriglyceridemia (TG ≥ 150 mg/dL)	3 (5.7%)	14 (25.5%)	0.012
Hyperuricemia (>6 mg/dL)	5 (9.4%)	14 (25.5%)	0.065
IGF (100–125 mg/dL)	3 (5.7%)	9 (16.4%)	0.163
IGT (140–199 mg/dL)	4 (7.5%)	10 (18.2%)	0.236
HOMA-IR (>2.5)	6 (11.3%)	19 (34.5%)	0.010

ALT—alanine transaminase; AST—aspartate aminotransferase; LDL-C—low-density lipoprotein cholesterol; HDL—high-density lipoprotein cholesterol; TG—triglycerides; IGF—impaired glucose fasting; IGT—impaired glucose tolerance; HOMA-IR—Homeostatic Model Assessment for Insulin Resistance; df = 1.

**Table 4:** Loadings of the first three primary components.

Parameter	PC1 (General Body Size)	PC2 (Height and Age)	PC3 (Body Fat Distribution: Visceral and/or in Extremities)
Age [years]	0.10	**0.50**	0.11
Height [cm]	0.03	**−0.54**	−0.18
Weight [kg]	**0.35**	−0.10	−0.06
BMI [kg/m^2^]	**0.34**	0.10	−0.00
LBM [kg]	**0.31**	−0.27	−0.14
PBF [%]	**0.30**	0.21	0.19
MBF [kg]	**0.35**	0.01	0.04
TBW [kg]	**0.29**	−0.25	−0.27
VFL	**0.27**	0.19	**0.41**
Waist [cm]	**0.33**	0.13	−0.26
Hip [cm]	**0.33**	−0.09	0.19
WHR	0.16	0.33	**−0.62**
Average leg volume [mL]	0.20	−0.32	**0.40**

BMI—body mass index; LBM—lean body mass; PBF—percentage body fat; MBF—mass of body fat; TBW—total body water; VFL—visceral fat level; WHR—waist/hip ratio; Top variables for each component are bolded.

**Table 5:** Averaged RVI for the predictors across all target variables.

Parameter	Sum of Weights
Disease type	0.65
PC1	0.68
PC2	0.52
PC3	0.71
PC1 × Disease type	0.24
PC2 × Disease type	0.11
PC3 × Disease type	0.29

PC1—general body size; PC2—height and age; PC3—body fat distribution: visceral and/or in extremities.

**Table 6:** Direction of estimated effects for the averaged models.

	ALT	LDL-C	HDL-C	Uric Acid	TG	Glucose 0 min	Glucose 120 min	HOMA-IR
Disease (overweight/obesity)	+	+		+		+	+	+
PC1				+	+	+	–	+
PC2		+					+	+
PC3	+	–	–	+	–	–		
PC1 × Disease							+	+
PC2 × Disease								
PC3 × Disease	–	–				+		

ALT—alanine transaminase; LDL-C—low-density lipoprotein cholesterol; HDL—high-density lipoprotein cholesterol; TG—triglycerides; HOMA-IR—Homeostatic Model Assessment for Insulin Resistance; PC1—general body size; PC2—height and age; PC3—body fat distribution: visceral and/or in extremities. Note: only high-importance predictors (RVI>0.6) shown. ‘+’ sign indicates a greater change in metabolic alterations, while ‘–’ indicates a lower chance of metabolic alterations.

## Data Availability

All data used to support the findings of this study are available from the corresponding author upon reasonable request due to the privacy of patients.

## Supplementary Materials

The following supporting information can be downloaded at: https://www.mdpi.com/article/10.3390/biomedicines13040867/s1. Figure S1: Scree plot presenting variances of eigenvalues fitted using Principal Component Analysis. Table S1: Variances of all principal components and variance explained by those components; Table S2: A set of best-fitted models describing each target variable; Table S3: Averaged model describing each target variable.
