# Supplementary material for: Metabolic Alterations in Women with Lipedema Compared to Women with Lifestyle-Induced Overweight/Obesity
Source: Biomedicines. 2025 Apr 3;13(4):867. doi: 10.3390/biomedicines13040867 (PMC12024828; doi:10.3390/biomedicines13040867)
Supplement: Supplementary file 1 [file biomedicines-13-00867-s001.zip › biomedicines-3553738-supplementary.pdf]

biomedicines

MDPI

SUPPLEMENT

Figure S1. Scree plot presenting variances of eigenvalues fitted using Principal Component Analysis.

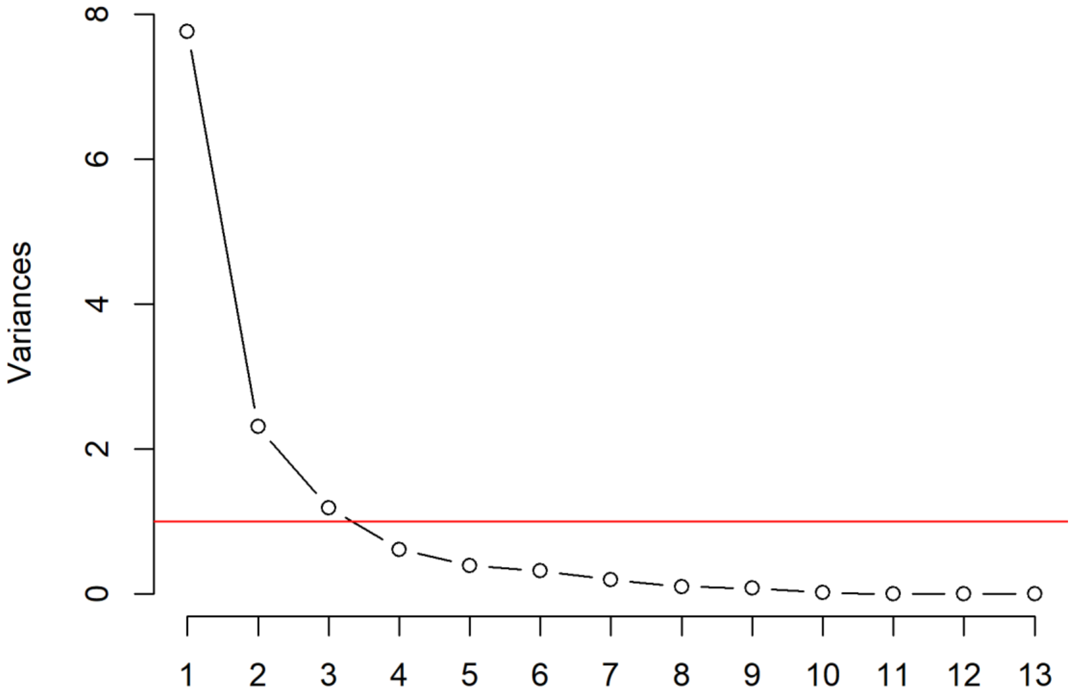

Note: Red horizontal line presents variance with a value of 1.

Table S1. Variances of all principal components and variance explained by those components.

Component Name	Component Variance	Variance explained by Component	Cumulative variance explained
PC1	7.76	0.60	0.60
PC2	2.31	0.18	0.77
PC3	1.19	0.09	0.87
PC4	0.62	0.05	0.91
PC5	0.40	0.03	0.94
PC6	0.32	0.02	0.97
PC7	0.20	0.02	0.98
PC8	0.10	0.01	0.99
PC9	0.08	0.01	1.00
PC10	0.02	<0.01	1.00
PC11	<0.01	<0.01	1.00
PC12	<0.01	<0.01	1.00
PC13	<0.01	<0.01	1.00

Table S2. A set of best-fitted models describing each target variable.

S2.ALT

		Model 1	Model 2	Model 3
Intercept	Estimate	−4.00	−3.89	−4.02

	p-value	0.0005*	0.0006*	0.0005*
Disease type	Estimate	2.17	1.95	2.14
	p-value	0.0793	0.1212	0.0879
PC1	Estimate	-	0.30	-
	p-value	-	0.3696	-
PC2	Estimate	-	-	0.16
	p-value	-	-	0.6334
PC3	Estimate	1.52	1.43	1.55
	p-value	0.0266*	0.0391*	0.0263*
PC1 * Disease type	Estimate	-	-	-
	p-value	-	-	-
PC2 * Disease type	Estimate	-	-	-
	p-value	-	-	-
PC3 * Disease type	Estimate	−1.96	−1.88	−2.02
	p-value	0.0194*	0.0254*	0.0183*
AICc		77.4	78.8	79.4
Delta		0.00	1.41	1.99
Weight		0.536	0.265	0.199
R-squared		0.070	0.088	0.065

S2.AST

		Model 1	Model 2	Model 3	Model 4	Model 5
Intercept	Estimate	−2.01	−1.93	−2.04	−1.96	−2.02
	p-value	<0.0001*	<0.0001*	<0.0001*	<0.0001*	<0.0001*
Disease type	Estimate	-	-	-	-	-
	p-value	-	-	-	-	-
PC1	Estimate	0.44	-	0.45	-	0.46
	p-value	0.1341	-	0.1354	-	0.1277
PC2	Estimate	-	-	-	-	0.17
	p-value	-	-	-	-	0.5689
PC3	Estimate	-	-	0.27	0.27	-
	p-value	-	-	0.3579	0.3537	-
PC1 * Disease type	Estimate	-	-	-	-	-
	p-value	-	-	-	-	-
PC2 * Disease type	Estimate	-	-	-	-	-
	p-value	-	-	-	-	-
PC3 * Disease type	Estimate	-	-	-	-	-
	p-value	-	-	-	-	-
AICc		80.0	80.1	81.2	81.4	81.8
Delta		0.00	0.19	1.28	1.41	1.80
Weight		0.300	0.273	0.158	0.148	0.122
R-squared		0.019	0.000	0.025	0.014	0.018

S2.LDL-C

		Model 1	Model 2	Model 3
Intercept	Estimate	0.01	0.35	0.08
	p-value	0.9786	0.1046	0.8353
Disease type	Estimate	0.22	-	0.22

	p-value	0.6571	-	0.6647
PC1	Estimate	-	-	-
	p-value	-	-	-
PC2	Estimate	0.58	0.57	0.84
	p-value	0.0140*	0.0144*	0.0153*
PC3	Estimate	−0.07	−0.55	−0.09
	p-value	0.8230	0.0150*	0.7850
PC1 * Disease type	Estimate	-	-	-
	p-value	-	-	-
PC2 * Disease type	Estimate	-	-	−0.56
	p-value	-	-	0.2528
PC3 * Disease type	Estimate	−1.13	-	−1.02
	p-value	0.0422*	-	0.0728
AICc		131.9	132.3	132.9
Delta		0.00	0.36	0.92
Weight		0.406	0.339	0.256
R-squared		0.172	0.123	0.175

S2.HDL-C p.1

		Model 1	Model 2	Model 3	Model 4	Model 5
Intercept	Estimate	−3.57	−2.81	−2.61	−2.89	−2.70
	p-value	0.0006*	<0.0001*	<0.0001*	<0.0001*	<0.0001*
Disease type	Estimate	0.07	-	-	-	-
	p-value	0.9625	-	-	-	-
PC1	Estimate	-	-	-	0.44	0.44
	p-value	-	-	-	0.2690	0.2540
PC2	Estimate	-	-	-	-	-
	p-value	-	-	-	-	-
PC3	Estimate	0.56	−0.70	-	−0.68	-
	p-value	0.4627	0.1139	-	0.1198	-
PC1 * Disease type	Estimate	-	-	-	-	-
	p-value	-	-	-	-	-
PC2 * Disease type	Estimate	-	-	-	-	-
	p-value	-	-	-	-	-
PC3 * Disease type	Estimate	−2.08	-	-	-	-
	p-value	0.0483*	-	-	-	-
AICc		52.4	52.4	53.1	53.3	53.8
Delta		0.00	0.00	0.68	0.92	1.47
Weight		0.182	0.182	0.129	0.115	0.087
R-squared		0.117	0.058	0.000	0.085	0.006

S2.HDL-C p.2

		Model 6	Model 7	Model 8	Model 9
Intercept	Estimate	−3.47	−3.16	−2.82	−2.99
	p-value	0.0009*	<0.0001*	<0.0001*	<0.0001*
Disease type	Estimate	−0.17	0.90	-	0.34
	p-value	0.9059	0.2986	-	0.7232
PC1	Estimate	0.37	-	-	-

	p-value	0.4139	-	-	-
PC2	Estimate	-	-	−0.23	-
	p-value	-	-	0.6092	-
PC3	Estimate	0.45	-	−0.70	−0.63
	p-value	0.5572	-	0.1148	0.2004
PC1 * Disease type	Estimate	-	-	-	-
	p-value	-	-	-	-
PC2 * Disease type	Estimate	-	-	-	-
	p-value	-	-	-	-
PC3 * Disease type	Estimate	−1.96	-	-	-
	p-value	0.0615	-	-	-
AICc		53.9	54.0	54.2	54.4
Delta		1.55	1.58	1.85	2.00
Weight		0.084	0.082	0.072	0.067
R-squared		0.133	0.011	0.065	0.060

S2.Uric acid

		Model 1	Model 2	Model 3	Model 4
Intercept	Estimate	−3.27	−3.30	−3.50	−3.59
	p-value	<0.0001*	<0.0001*	<0.0001*	<0.0001*
Disease type	Estimate	2.13	1.96	2.42	2.32
	p-value	0.0130*	0.0222*	0.0137*	0.0220*
PC1	Estimate	0.67	0.82	1.27	1.49
	p-value	0.0358*	0.0189*	0.1053	0.0889
PC2	Estimate	-	0.46	-	0.47
	p-value	-	0.1453	-	0.1479
PC3	Estimate	1.01	0.97	0.98	0.95
	p-value	0.0110*	0.0167*	0.0143*	0.0206*
PC1 * Disease type	Estimate	-	-	−0.74	−0.80
	p-value	-	-	0.3872	0.3879
PC2 * Disease type	Estimate	-	-	-	-
	p-value	-	-	-	-
PC3 * Disease type	Estimate	-	-	-	-
	p-value	-	-	-	-
AICc		78.9	79.0	80.3	80.5
Delta		0.00	0.08	1.41	1.54
Weight		0.343	0.329	0.170	0.158
R-squared		0.191	0.214	0.195	0.221

S2.TG

		Model 1	Model 2	Model 3
Intercept	Estimate	−2.17	−2.26	−2.49
	p-value	<0.0001*	<0.0001*	<0.0001*
Disease type	Estimate	-	-	0.57
	p-value	-	-	0.4741
PC1	Estimate	0.98	1.08	0.91
	p-value	0.0027*	0.0025*	0.0073*
PC2	Estimate	-	0.38	-

	p-value	-	0.2214	-
PC3	Estimate	−0.81	−0.84	−0.68
	p-value	0.0174*	0.0158*	0.0754
PC1 * Disease type	Estimate	-	-	-
	p-value	-	-	-
PC2 * Disease type	Estimate	-	-	-
	p-value	-	-	-
PC3 * Disease type	Estimate	-	-	-
	p-value	-	-	-
AICc		77.9	78.6	79.6
Delta		0.00	0.67	1.64
Weight		0.464	0.332	0.205
R-squared		0.160	0.174	0.167

S2.Glucose p.0

		Model 1	Model 2	Model 3	Model 4
Intercept	Estimate	−2.75	−2.55	−2.30	−2.41
	p-value	0.0006*	0.0003*	<0.0001*	<0.0001*
Disease type	Estimate	0.25	0.68	-	-
	p-value	0.8047	0.4246	-	-
PC1	Estimate	1.07	0.74	0.71	0.82
	p-value	0.0098*	0.0394*	0.0306*	0.0222*
PC2	Estimate	−0.40	-	-	0.48
	p-value	0.5072	-	-	0.1466
PC3	Estimate	−1.77	−1.55	-	-
	p-value	0.0417*	0.0533	-	-
PC1 * Disease type	Estimate	-	-	-	-
	p-value	-	-	-	-
PC2 * Disease type	Estimate	1.41	-	-	-
	p-value	0.0733	-	-	-
PC3 * Disease type	Estimate	2.23	2.23	-	-
	p-value	0.0315*	0.0220*	-	-
AICc		67.8	68.1	69.0	69.0
Delta		0.00	0.38	1.21	1.25
Weight		0.236	0.196	0.129	0.126
R-squared		0.218	0.133	0.059	0.080

S2.Glucose p. 60 min.

		Model 5	Model 6	Model 7
Intercept	Estimate	−2.54	−2.74	−2.87
	p-value	0.0003*	0.0011*	<0.0001*
Disease type	Estimate	0.50	0.97	0.38
	p-value	0.5610	0.3126	0.6679
PC1	Estimate	0.84	1.54	0.88
	p-value	0.0267*	0.1156	0.0192*
PC2	Estimate	0.38	-	−0.36
	p-value	0.2729	-	0.6065
PC3	Estimate	−1.53	−1.92	-

	p-value	0.0560	0.0503	-
PC1 * Disease type	Estimate	-	−0.96	-
	p-value	-	0.3606	-
PC2 * Disease type	Estimate	-	-	1.36
	p-value	-	-	0.1070
PC3 * Disease type	Estimate	2.13	2.59	-
	p-value	0.0288*	0.0203*	-
AICc		69.2	69.5	69.6
Delta		1.42	1.68	1.83
Weight		0.116	0.102	0.095
R-squared		0.154	0.123	0.142

S2.Glucose p. 120 min.

		Model 1	Model 2	Model 3	Model 4
Intercept	Estimate	−3.04	−4.21	−3.00	−4.04
	p-value	0.0003*	0.0098*	0.0008*	0.0125*
Disease type	Estimate	0.98	2.39	0.68	2.00
	p-value	0.2683	0.1606	0.4702	0.2509
PC1	Estimate	−1.25	−2.18	−1.26	−2.09
	p-value	0.0979	0.0840	0.1092	0.0955
PC2	Estimate	0.82	1.67	0.90	1.64
	p-value	0.0266*	0.0513	0.0202*	0.0559
PC3	Estimate	-	-	−0.42	−0.34
	p-value	-	-	0.2632	0.3780
PC1 * Disease type	Estimate	2.19	2.97	2.23	2.93
	p-value	0.0170*	0.0247*	0.0192*	0.0263*
PC2 * Disease type	Estimate	-	−1.23	-	−1.10
	p-value	-	0.2050	-	0.2638
PC3 * Disease type	Estimate	-	-	-	-
	p-value	-	-	-	-
AICc		75.9	76.2	76.9	77.8
Delta		0.00	0.31	1.01	1.87
Weight		0.350	0.300	0.212	0.138
R-squared		0.111	0.164	0.138	0.182

S2.HOMA-IR

		Model 1	Model 2	Model 3	Model 4	Model 5
Intercept	Estimate	−2.26	−2.11	−1.49	−2.20	−2.14
	p-value	<0.0001*	0.0002*	<0.0001*	<0.0001*	<0.0001*
Disease type	Estimate	1.15	0.78	-	1.29	1.22
	p-value	0.0706	0.2676	-	0.0363*	0.0344*
PC1	Estimate	−0.22	−0.15	0.94	−0.06	0.79
	p-value	0.7000	0.8056	0.0021*	0.8971	0.0099*
PC2	Estimate	0.56	0.60	0.52	-	0.44
	p-value	0.0462*	0.0354*	0.0485*	-	0.0986
PC3	Estimate	-	−0.39	−0.61	-	-
	p-value	-	0.2281	0.0291*	-	-
PC1 * Disease type	Estimate	1.43	1.39	-	1.04	-

	p-value	0.0484*	0.0616	-	0.0922	-
PC2 * Disease type	Estimate	-	-	-	-	-
	p-value	-	-	-	-	-
PC3 * Disease type	Estimate	-	-	-	-	-
	p-value	-	-	-	-	-
AICc		98.2	99.0	99.8	100.0	100.2
Delta		0.00	0.77	1.55	1.83	1.97
Weight		0.343	0.233	0.158	0.137	0.128
R-squared		0.239	0.239	0.188	0.200	0.215

Note: Stars indicate significant predictors at alpha = 0.05.

Table S3. Averaged model describing each target variable.

S3.ALT

Predictor	Estimate	Standard Error	*p*-value	RVI - N models	RVI - sum of weights
Disease type	3.17	1.88	0.0959	3	1
PC_1	0.9	1	0.3755	1	0.20
PC_2	0.49	1.02	0.6376	1	0.26
PC_3	4.51	2.07	0.0319	3	1
PC3 * Disease type	−3.57	1.55	0.0224	3	1

S3.AST

Predictor	Estimate	Standard Error	*p*-value	RVI - N models	RVI - sum of weights
PC_1	1.35	0.9	0.1379	3	0.58
PC_2	0.51	0.89	0.5736	1	0.12
PC_3	0.81	0.88	0.3617	2	0.31

S3.LDL-C

Predictor	Estimate	Standard Error	*p*-value	RVI - N models	RVI - sum of weights
Disease type	0.22	0.51	0.6641	2	0.66
PC_2	1.28	0.58	0.0291	3	1
PC_3	−0.48	0.75	0.5209	3	1
PC_2 * Disease type	−0.75	0.66	0.2588	1	0.26
PC_3 * Disease type	−1.35	0.7	0.0568	2	0.66

S3.HDL-C

Predictor	Estimate	Standard Error	*p*-value	RVI - N models	RVI - sum of weights
Disease type	0.45	2.59	0.8638	4	0.41
PC_1	1.64	1.61	0.315	3	0.29
PC_2	−0.89	1.74	0.6136	1	0.07
PC_3	−0.9	3.29	0.7858	6	0.70
PC_3 * Disease type	−4.94	2.55	0.0555	2	0.27

S3.Uric acid

Predictor	Estimate	Standard Error	*p*-value	RVI - N models	RVI - sum of weights
Disease type	2.94	1.26	0.021	4	1
PC_1	2.58	1.71	0.1342	4	1
PC_2	1.27	0.87	0.1514	2	0.49
PC_3	2.6	1.06	0.0161	4	1
PC_1 * Disease type	−1.48	1.72	0.3942	2	0.33

S3.TG

Predictor	Estimate	Standard Error	*p*-value	RVI - N models	RVI - sum of weights
Disease type	0.78	1.09	0.4796	1	0.20
PC_1	2.73	0.95	0.0043	3	1
PC_2	1.04	0.85	0.2272	1	0.33
PC_3	−2.18	0.98	0.028	3	1

S3.Glucose p.0

Predictor	Estimate	Standard Error	*p*-value	RVI - N models	RVI - sum of weights
Disease type	0.83	1.54	0.593	5	0.74
PC_1	2.98	1.7	0.0828	7	1
PC_2	−0.14	2.13	0.9493	4	0.57
PC_3	−5.43	2.8	0.0554	4	0.65
PC_1 * Disease type	−2.18	2.39	0.3666	1	0.10
PC_2 * Disease type	3.01	1.74	0.0866	2	0.33
PC_3 * Disease type	4.47	2.03	0.0297	4	0.65

S3.Glucose p. 120 min.

Predictor	Estimate	Standard Error	*p*-value	RVI - N models	RVI - sum of weights
Disease type	2.04	2.07	0.3303	4	1
PC_1	−4.48	3.01	0.1415	4	1
PC_2	3.31	2.07	0.1126	4	1
PC_3	−1.12	1.1	0.3156	2	0.35
PC_1 * Disease type	5.07	2.36	0.034	4	1
PC_2 * Disease type	−2.15	1.76	0.2302	2	0.44

S3.HOMA-IR

Predictor	Estimate	Standard Error	*p*-value	RVI - N models	RVI - sum of weights
Disease type	1.27	0.79	0.1118	4	0.84
PC_1	0.31	1.63	0.8519	5	1
PC_2	1.28	0.66	0.0558	4	0.86

PC_3	−1.12	0.76	0.1449	2	0.39
PC_1 * Disease type	2.22	1.2	0.0683	3	0.71

Note: Estimated coefficients are based on the weighted average from best-fitted models containing given predictor. RVI (relative variable importance) indicates the number and total weight of best-fitted models containing given predictor.
